# Oral Ferric Maltol Does Not Adversely Affect the Intestinal Microbiome of Patients or Mice, but Ferrous Sulphate Does

**DOI:** 10.3390/nu13072269

**Published:** 2021-06-30

**Authors:** Awad Mahalhal, Alessandra Frau, Michael D. Burkitt, Umer Z. Ijaz, Christopher A. Lamb, John C. Mansfield, Stephen Lewis, D. Mark Pritchard, Chris S. Probert

**Affiliations:** 1 Department of Molecular and Clinical Cancer Medicine, Institute of Systems, Molecular and Integrative Biology, University of Liverpool, Liverpool L69 3GE, UK; A.Frau@liverpool.ac.uk (A.F.); dmpritch@liverpool.ac.uk (D.M.P.); mdcsjp@liverpool.ac.uk (C.S.P.); 2 Division of Diabetes endocrinology and Gastroenterology, Faculty of Biology Medicine and Health, University of Manchester, Manchester M13 9PL, UK; michael.burkitt@manchester.ac.uk; 3 School of Engineering, University of Glasgow, Glasgow G12 8QQ, UK; Umer.Ijaz@glasgow.ac.uk; 4 Translational and Clinical Research Institute, Faculty of Medical Sciences, Newcastle University, Newcastle upon Tyne NE2 4HH, UK; christopher.lamb@newcastle.ac.uk; 5 Department of Gastroenterology, Newcastle upon Tyne Hospitals NHS Foundation Trust, Newcastle upon Tyne NE1 4LP, UK; john.mansfield8@btinternet.com; 6 Department of Gastroenterology, Derriford Hospital, Plymouth PL6 8DH, UK; sjl@doctors.org.uk

**Keywords:** iron, microbiome, dysbiosis

## Abstract

Background and Aims: Altering dietary ferrous sulphate (FS) consumption exacerbates a murine model of colitis and alters the intestinal microbiome. We investigated the impact of oral ferric maltol (FM) and FS on mice with dextran sodium sulphate (DSS) induced colitis, and the microbiome of patients with iron deficiency. Methods: Mice had acute colitis induced, with 2% DSS for 5 days, followed by water. During this period, groups of mice were fed standard chow (200 ppm iron, SC, *n* = 8), or SC with 200ppm FS supplementation (*n* = 16, FSS), or SC with 200 ppm FM supplementation (*n* = 16, FMS). Clinical, pathological and microbiome assessments were compared at days 1 and 10. Fecal bacterial gDNA was extracted and the microbiome assessed by sequencing. Statistical inferences were made using MacQIIME. Principal Coordinates Analysis were used to visualize beta-diversity cluster analysis. Ten patients with IDA were treated with FS, and six with inactive inflammatory bowel disease received FM, supplements for four weeks: pre- and mid-treatment fecal samples were collected: the microbiome was assessed (see above). Results: In mice, after DSS treatment, there was a decrease in many genera in the SC and FSS groups: Lactobacillales increased in mice that received FMS. In humans, FS treatment led to an increase in five genera, but FM was not associated with any measurable change. The severity of DSS-induced colitis was greater with FSS than FMS. Conclusions: This study demonstrates differential and unique influences of ferric maltol and ferrous sulphate supplements on intestinal microbiota. These differences might contribute to the different side effects associated with these preparations.

## 1. Introduction

Traditional oral ferrous preparations, such as ferrous sulphate (FS), are associated with gastrointestinal disturbance such a cramp, diarrhoea, and constipation, especially in patients with inflammatory bowel disease (IBD). Side effects are thought to arise, at least in part, from the generation of free radicals, and lead to discontinuation of oral iron preparations in approximately 20% of patients [1,2,3,4]. Patients with IBD are prone to iron deficiency, and their disease relapses are associated with diarrhoea. These patients have co-existing diarrhoea and iron deficiency, and treatment with oral ferrous preparations may exacerbate diarrhoea and complicate the clinical picture [5]. Iron absorption is regulated by hepcidin. Hepcidin reduced when iron stores are depleted. However, inflammation increases hepcidin and this reduces absorption of iron [6,7]. Consequently, oral ferrous preparations may be inadequately absorbed in the presence of active IBD [8]. This has led to the development, and promotion, of intravenous iron therapies in this condition [9]. European Crohn’s and Colitis Organisation Guidelines promote the use of intravenous iron replacement for patients with severe anemia and/or active IBD, and oral therapies for patients with inactive IBD [10].

It is desirable to manage anaemic patients in the community and avoid intravenous therapy where possible as oral therapy, at home, is more convenient for patients and it costs less that intravenous therapy in hospitals [11]. Ferric maltol (FM) is a new form of oral iron supplementation that is licensed for use in patients, including those with IBD, who are iron deficient. Ferric maltol does not contain ferrous iron: instead, it has ferric iron that is sequestered by a sugar complex; most of this iron is absorbed in the small intestine, and unabsorbed iron remains sequestered, limiting the amount of free iron in the colon. In registration studies, ferric maltol was well-tolerated and without risk of relapse of IBD [12].

Intestinal bacteria have evolved to scavenge iron from their hosts. Moreover, hosts have adapted to limit the access to iron by bacteria [13]. In the presence of iron supplementation, there is potential to exceed the absorptive capacity (10–20 mg/day) [2] and the unabsorbed iron becomes available to colonic bacteria, which promotes the growth of some species [14]. There is evidence of a change in the intestinal microbial community (dysbiosis) following oral ferrous preparations in children with anemia [15,16]. A change in the intestinal microbiome may contribute to the side effects of iron supplementation and the risk of relapse of IBD.

We have, therefore, investigated the effect of ferric iron supplementation on a murine model of colitis and also the intestinal microbiome of patients with iron deficiency.

## 2. Materials and Methods

### 2.1. Murine Experiments

In this study we did order 40 wild-type C57BL/six female mice, aged 8–9 weeks old from Charles River Laboratories (Margate, UK) where each mouse was caged individually. Three altered iron diets were administered, and we collected faecal samples pre and post DSS treatment. First group was control mice (*n* = 8) were fed Rat and Mouse Breeder and Grower Pelleted CRM (P) which contains 200 ppm ferrous sulphate, considered as a standard chow (SC). The other two modifications of the standard chow were 200 ppm ferrous sulphate supplemented (FSS) standard chow (*n* = 16), and 200 ppm ferric maltol supplemented (FMS) standard chow (*n* = 16). The care of, and experimentation on, mice was carried out in accordance with the UK Home Office regulations.

In order to induce colitis chemically, 2% of dextran sulfate sodium (DSS) was used. This chemical induction of colitis was popular because of its simplicity and its many similarities with human ulcerative colitis [17]. Diet was introduced on the same day that the mice were given a 2% solution of DSS (M.W. 36,000–50,000Da; Catalogue number: 160110; Lot number: 6683K; MP Biomedicals, LLC, UK) to drink for five days to induce colitis (~150 mL/mouse over 5 days), followed by another 5 days of DSS-free water. On day 10 measured from the start of the experiment, all mice were euthanised.

The entire colon was detached and only the distal portion was fixed, and wax embedded. Then about 4 μm sections were stained with haematoxylin and eosin, and inflammation was assessed by histology by the first author (AM), while blinded to the dietary treatment. Colitis parameters such as inflammatory cell infiltrate and tissue damage were recorded as described by Bauer et al. [18].

At two different time points, day 1 and 10, faecal pellets were collected from the cage of each mouse separately. Then faecal iron concentration was measured using an iron immunoassay kit (MAK025, Sigma-Aldrich), with an acidic buffer, where total iron (Fe^2+^ and Fe^3+^) was measured. However, the difference between samples taken at different time points reflects both bleeding, from the colon in DSS-treated animals, as well as unabsorbed oral iron supplements.

Bacterial DNA was extracted from ~20 mg of the faecal samples using Startec Kit [PSP^®^ Spin Stool DNA Plus Kit] according to the supplier’s protocol. The V4 region of extracted DNA was amplified by PCR: the products were quantified by Qubit^®^ 2.0 Fluorometer (Invitrogen) and visualised on an agarose gel. The V4 primers described by Caporaso et al. [19] were used (Forward: F515: 5′-GTG-CCA-GCM-GCC-GCG-GTA-A-3′, and Reverse: 806R: 5′-GGA-CTA-CNN-GGG-TNT-CTA-AT-3′), a KAPA HiFi Hot start Ready Mix PCR Kit (Kapa Biosystems, Boston, Massachusetts, United States). Subsequently, 16S metagenomic sequencing was performed at the Centre for Genomic Research, as described previously [17]. The DNA was sequenced as above.

### 2.2. Human Work

We undertook parallel studies of the intestinal microbiome of (i) six patients with quiescent inflammatory bowel disease (Crohn’s disease *n* = 5, Harvey Bradshaw Index ≤ 4; ulcerative colitis *n* = 1, Simple Colitis Activity Index ≤ 2), with iron deficiency, taking ferric maltol 30 mg bd and (ii) 10 patients with iron deficiency without IBD taking ferrous sulphate 200 mg bd, as part of routine clinical care, in two different NHS Trusts (Newcastle upon Tyne Hospitals NHS Foundation Trust and University Hospitals Plymouth NHS Trust, Plymouth, Devon, UK). Approval was granted for human stool collection in Newcastle upon Tyne Hospitals NHS Foundation Trust by the Newcastle Biobank (North East—Newcastle & North Tyneside 1 Research Ethics Committee Ref 17/NE/0361) and for University Hospitals Plymouth NHS Trust by UK NHS Health Research Authority’s Research Ethics Service (RES) Committee South West—Central Bristol (REC reference 14/SW/1162). All patients gave written informed consent for sample collection. Iron deficiency was defined according to local laboratory references ranges. Patients were asked to donate a faecal sample before commencing oral iron replacement and 4 weeks later, while taking therapy. Each patient donated a single spoon of faeces, passed on the morning of their clinical assessment: if patients had unpredictable patterns of defecation, they collected the sample on the day before the clinic, and kept the sample in a −20 °C freezer overnight. About 20 g of faeces was stored at −80 °C at the recruiting hospital and then sent to the University of Liverpool on dry ice.

Bacterial DNA was extracted from human faecal samples using the Startec Kit (PSP^®^ Spin Stool DNA Plus Kit). The DNA was sequenced as above.

### 2.3. Statistics

Normally distributed data were assessed by analysis of variance followed by multiple comparisons; other data were assessed by Kruskal-Wallis test (Stats Direct version 3.0.171) [17]. A significant difference was defined as *p* < 0.05.

For the bioinformatic analysis of microbiota data, the relative abundance of phyla before and after supplementation in each group was reported. OTUs were filtered at a 0.05% abundance threshold [20]. Alignment and phylogeny analyses were carried out on MacQIIME (v1.9.1) [21] with default algorithms (PyNAST [22] and FastTree2 [23]). Microbial data were analysed with R (version 3.6.3) [24] using scripts created by the authors [25]. The Vegan package [26] was used for alpha and beta diversity analysis. For beta diversity, distance measures (Bray-Curtis, unweighted and weighted UniFrac [27] were calculated using Phyloseq [28]. Principal Coordinates Analysis (PCoA) were used to visualize beta-diversity cluster analysis. Permutational multivariate analysis of variance (PERMANOVA) of sources of variations (groups in this study) against the distance matrices as mentioned above were performed with Vegan’s adonis function (permutations = 999). Pair-wise ANOVA was calculated with aov [29]. DESeq2 was used (DESeqDataSetFromMatrix) using default parameters, to carry out taxa differential analysis, results with a significance value cut-off of 0.05, adjusted for multiple comparisons, and fold-change of at least 2 were kept. Within taxa, differential analysis, to rank in terms of importance of each significant taxa, a random forest classifier was used; the random Forest function [30] was used to make this calculation. The importance function was used to extract Mean Decreased Accuracy (MDA) and Mean Decreased Gini (MDG).

All authors had access to the study data and had reviewed and approved the final manuscript.

## 3. Results

### 3.1. Murine Results

#### 3.1.1. Clinical Data

**Figure 1:**
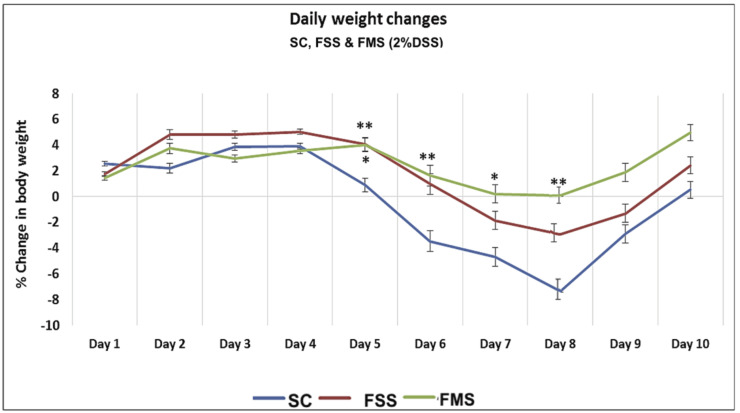
Percentage of weight change associated with acute DSS in mice receiving standard chow (SC) and chow supplemented with ferrous sulphate (FSS) or ferric maltol (FMS). Data are presented as a mean ± standard error of the mean. Statistical differences were assessed by Kruskal–Wallis test followed by Dunn’s multiple comparison tests (* *p* < 0.05, ** *p* < 0.01). (*n* = 40 ♀ mice) (asterisks above FMS vs. SC, asterisk below FSS vs. SC).

Acute colitis was induced in all three groups of mice (SC, FSS and FMS): weight loss was seen in the SC and FSS groups from day 5 with the maximal loss occurring on day 8. The weight loss was least in mice receiving ferric maltol (*p* = 0.01) (Figure 1).

**Figure 2:**
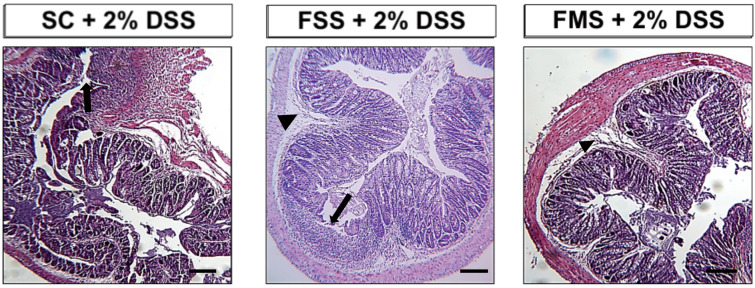
Representative haematoxylin- and eosin-stained sections of the distal colon from mice treated with 2% *w/v* dextran sodium sulphate for 5 days followed by another 5 days on plain drinking water. Arrowheads highlight submucosal oedema; arrows highlight almost complete loss of colonic epithelium. Scale Bar: 200 µm.

At necropsy, all mice had histological features of colitis (Figure 2) which were significantly worse in mice that had received ferrous sulphate supplementation.

**Figure 3:**
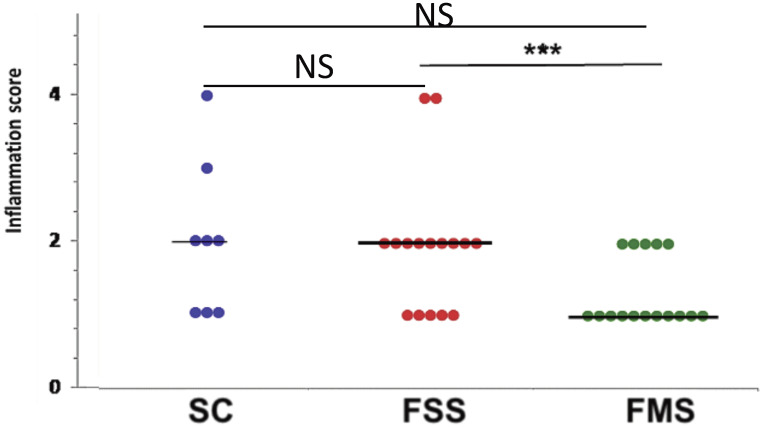
Spread plot of inflammation (colitis) scores for each group of mice at necropsy, at day 10. Horizontal lines at the median. Differences tested by one-way ANOVA (overall *p* = 0.06) followed by multiple comparisons Dunn’s test (*** *p* < 0.001). (NS non-significant).

Histological colonic inflammation severity scores in the FSS group were significantly greater than those observed in mice ingesting standard chow (SC) or FMS, at day 10 (Figure 3).

**Figure 4:**
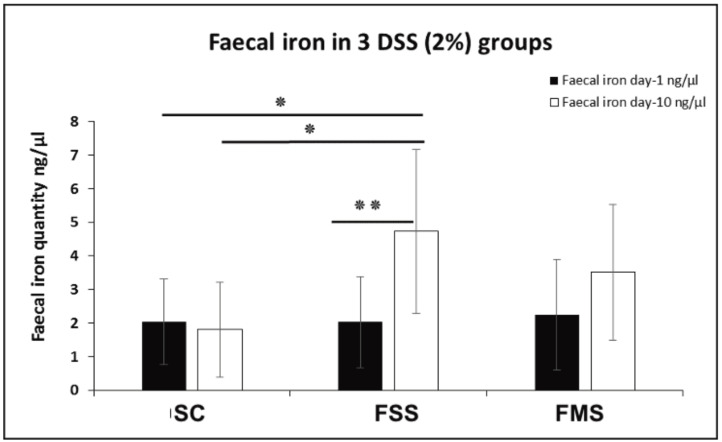
Faecal iron concentration at two different time points (day 1 and 10) for three DSS-treated groups of mice (SC, FSS and FMS diets). Data are presented as a mean ± standard error of the mean. Differences were tested by t-test (inter-comparison) and by one-way ANOVA (intra-comparison) followed by post hoc test. (* *p* < 0.05, ** *p* < 0.01).

There was no difference in faecal iron concentration between the three experimental groups at baseline (day 1). At day 10 there was a significant increase in faecal iron compared to baseline in the mice receiving the FSS diet; the change with FMS was not statistically significant. This is consistent with the greater severity of colitis in mice receiving the FSS diet (Figure 4).

#### 3.1.2. Bacterial Diversity Data Analysis at Phylum Level for Murine Experiments

**Figure 5:**
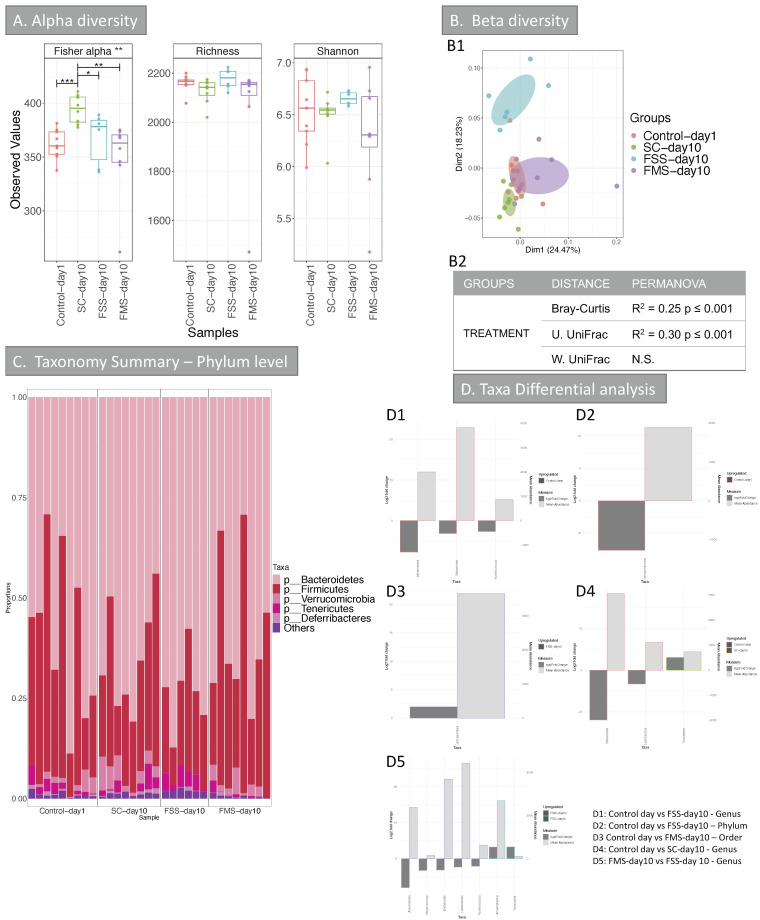
Alpha and Beta diversity, and taxonomy results of the intestinal microbiome (mice experiment). (**A**) Alpha diversity (OTUs level) of the intestinal microbiome from the 4 groups: Control-day 1, SC-day 10, FSS-day 10 and FMS-day 10. Three indices were considered: Fisher alpha (a parametric index that models species’ abundance as logseries distribution), richness (number of species) and Shannon index (a widely used index that considers species’ abundance and evenness). Pair-wise ANOVA was calculated between the groups and if significant, stars are shown on top (* *p* < 0.05, ** *p* < 0.01 and *** *p* < 0.001). (**B**) Beta diversity results; (**B1**) Principal Co-ordinate Analysis (PCOA) showing clustering of samples. The chart was produced using unweighted UniFrac (UniFrac) at OTUs level. Ellipses are 95% confidence interval of standard error. The table in (**B2**) summarises PERMANOVA results for all the distances, Bray-Curtis, unweighted UniFrac (U. UniFrac) and weighted (W. UniFrac). R2 refers to the percentage of variability among samples’ microbiome that can be explained by that factor/metadata. (**C**) Taxonomy summary for stool at phylum level. (**D**) Taxa differential analysis at phylum, order and genus levels are presented through bar charts; these show Log2 fold change between the groups compared, (*y* axis on the left and dark grey bar) and the mean abundance across all the samples (*y* axis on the right and light grey bar), details of the comparisons per chart are in the right bottom of the chart. Detail of taxa differential analysis results, including p values and adjusted *p* values, is in Supplementary Table S3. FS = ferrous sulphate and FM = ferric maltol.

11,945,462 chimera-checked *16S* rRNA sequences (145,676 ± 98,121 per sample) spanning 112,383 OTUs were obtained. All the samples were analysed; however, most of the baseline, pre-DSS treatment (day 1) samples of mice treated with SC and FSS (except for one sample) did not yield enough reads, so these were therefore discarded. The reason for this was not found. Consequently, FMS samples at day 1 and the one FSS sample at day 1 were used as day 1 controls in the analysis. The results are shown in Figure 5. Analysis of alpha diversity (Figure 5A) indicated that there was a reduction in Fisher alpha index in faecal samples taken from mice treated with FSS or FMS and the control at day 1 compared to SC day 10. Neither of the other two indices tested gave any significant result.

Clustering analysis shows the separation of mice treated with FSS from the other three categories, at day 10, after DSS treatment. Two factors were taken into account during the analysis, the diet treatment and mice ID; the second was not significant in explaining the diversity and was therefore discarded from the analysis. The diet treatment explains up to 30% of the diversity (Figure 5B2). Taxonomy analysis showed a reduction in *Verrucomicrobia* in mice treated with FSS post DSS (Figure 5C); this was confirmed with taxa differential analysis (Figure 5D), when this group was compared with control day 1 mice. A reduction in *Bacteroides*, *Akkermansia* and *Ruminococcus* were observed in FSS post DSS mice (FSS day 10). A reduction in *Bacteroides* was also observed, when comparing SC-day 10 with control day 1 mice, suggesting that this change may be related to the DSS treatment rather than the FS supplement per se. When comparing FMS day 10 mice with control at day 1 mice, significant results were observed only at order and OTU levels, both indicating an increase of *Lactobacillus* post-treatment, suggesting that FM supplement has a less dramatic effect in the microbiome compared to FS. This was confirmed when comparing FMS treated mice at day 10 to FSS treated mice at day 10,#; the first group showed an increase of group found at day 1 (e.g., Akkermansia, *Bacteroides*, *Ruminococcus* and *Lactobacillus*) compared to FSS day 10 mice. This last group showed an increase in *Anaeroplasma* and *Turicibacter*.

### 3.2. Human Results

The clinical and demographic features of the participants are summarised in Supplementary Tables S1 and S2.

**Figure 6:**
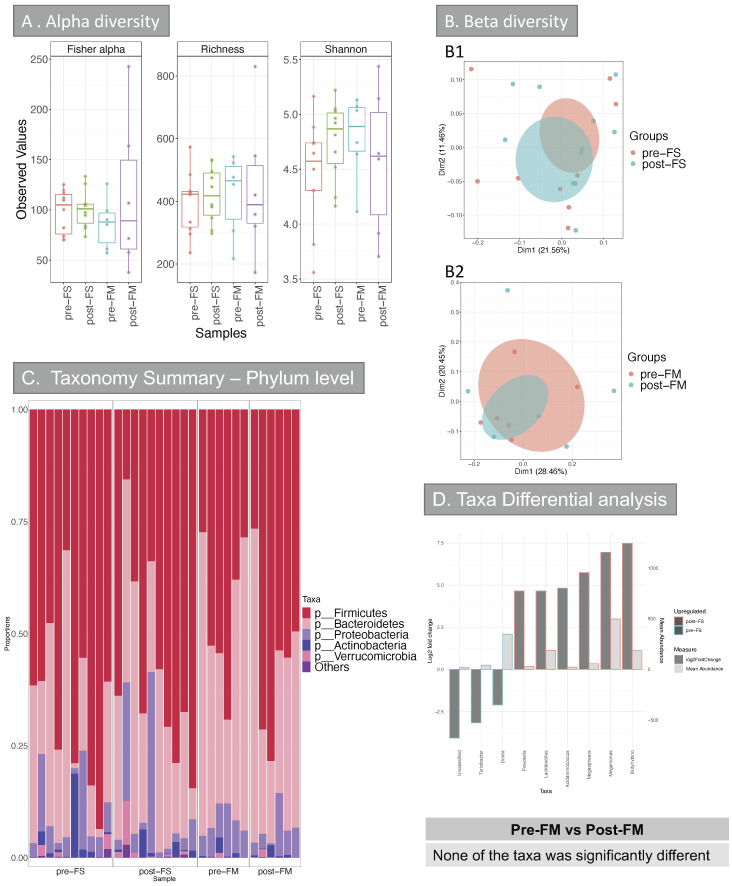
Alpha and Beta diversity, and taxonomy results of the intestinal microbiome (Human cohort). (**A**) Alpha diversity (OTUs level) of the intestinal microbiome from the 4 groups: pre-FS, post-FS, pre-FM, post-FM. Three indices were considered: Fisher alpha (a parametric index that models species’ abundance as logseries distribution), richness (number of species) and Shannon index (a widely used index that considers species’ abundance and evenness). Pair-wise ANOVA was calculated between the groups, but none was significant. (**B**) Beta diversity results (**B1/B2**) Principal Co-ordinate Analysis (PCOA) showing c––lustering of samples. The chart was produced using unweighted UniFrac (UniFrac) at OTUs level. Ellipses are 95% confidence interval of standard error. PERMANOVA analysis comparing the groups for all the distances (Bray-Curtis, unweighted UniFrac (UniFrac) and weighted (W. UniFrac)) were not significant. (**C**) Taxonomy summary at phylum level. (**D**) Taxa differential analysis at genus level are presented through bar charts, which show Log2 fold change between pre-FS vs. post-FS samples, (*y* axis on the left and dark grey bar) and the mean abundance across all the samples (*y* axis on the right and light grey bar). The comparison of samples from patients that were given FM before and after treatment (pre-FM vs. post-FM) did not give any significant results at any of the taxonomical levels analysed (phylum, class, order, family, genus and OTUs). Detail of taxa differential analysis results, including p values and adjusted p values, is in Supplementary Table S3. FS = ferrous sulphate and FM = ferric maltol.

Evaluation of the richness in species (alpha diversity) did not show any significant difference groups (Figure 6A).

Between sample variation (beta diversity) analysis (Figure 6B1,B2) showed that the main factor influencing the variability was the patient ID and the diagnosis (IBD vs. non-IBD). However, the iron treatment did not explain any of the diversity, also confirmed by the poor clustering (Figure 6B).

Taxa differential analysis did not show any significant difference in taxa at any level when comparing patients that were given FM pre- and post-treatment (Figure 6D), suggesting that none of the taxa was influenced by FM supplement. However, when comparing patients pre- and post-FS treatment, many genera were significantly different between the two categories (Figure 6D). Specifically, patients that underwent FS treatment showed a decrease of *Dorea* and *Turicibacter*. Meanwhile, many Firmicutes genera were increased in patients after taking FS (*Butyrivibrio*, *Megamonas*, *Megasphaera*, *Lactobacillus* and *Acidaminococcus*). Interestingly, these results differ from what was observed in mice, where *Turicibacter* was increased in FSS treated mice and *Lactobacillus* decreased in this group.

## 4. Discussion

Standard oral iron replacement is based on ferrous preparations, such as ferrous sulphate: unfortunately, these preparations are associated with gastrointestinal side effects (2). Ferric maltol was developed as an alternative oral iron supplement: it is licensed for the treatment of iron deficiency (EMA, March 2018). Ferric maltol appears to have fewer such side effects.

We undertook the first investigation of the effect of ferrous sulphate and ferric maltol on the microbiome. Initially, we investigated the effect of ferrous and ferric supplements on the development of DSS-induced colitis and the associated microbiome. All three groups of mice had similar baseline characteristics. There was clear impact on the severity of the DSS-colitis in mice that received supplemental ferrous sulphate assessed clinically (weight loss) and by histology. This may be a consequence of the dysbiosis or an effect of free radicals. We have previously reported that changes in oral ferrous iron are associated with worse DSS-induced colitis [17]. We are unable to conclude whether this difference is due to the effect of free radical on the mucosa, or to the dysbiosis.

Changing the form of oral iron supplementation also appeared to influence the community structure of the intestinal microbiome. The microbiome was assessed 5 days after treatment with DSS. There was an obvious clustering between mice treated with FS supplementation post DSS compared to the other three categories (Figure 5B1,B2). Deeper taxonomy analysis showed an increase in *Verrucomicrobia* in mice treated with FS post DSS (Figure 5C,D): others have reported this change in DSS colitis [30]. However, we only observed this change in the DSS-treated mice receiving the FSS diet. There was a reduction in three genera, including *Bacteroidetes*. The control mice taking the SC diet also showed a reduction in *Bacteroidetes*. This suggests that DSS-colitis may influence *Bacteroidetes.* The two other genera that were reduced after DSS did not change significantly with SC, implying the FS supplementation was a factor. *Turicibacter* was reduced after DSS in the SC group: this change has been reported previously [31,32].

In contrast, the only statistically significant change observed in the FMS treated mice, after DSS, was an increase in Lactobacillales: this has not been reported before. Pereira et al. reported that a nano ferric preparation leads to a relative increase the growth of Lactobacilli in mice [33].

It is interesting to note that when used as a probiotic, Lactobacilli ameliorate DSS colitis mice and rats [34,35]. Ferrous preparations are known to suppress the growth of Lactobacilli [13] and it appears that ferric and ferrous preparations have opposing effects on the growth of Lactobacilli

In this study, changes in dietary iron type and amounts therefore appeared to influence colonic inflammation in a DSS mouse model of inflammatory bowel disease. There appeared to be synergistic effects between iron and DSS on colonic inflammation. Inflammation, as well as oral iron, increases faecal iron concentrations [36,37], which can explain the greater increase in the faecal iron concentration in the FS group, compared to the other groups, despite equal concentrations of iron in the diets of FSS and FMS supplemented groups.

A single 200 mg tablet of ferrous sulphate contains 65 mg of elemental iron, which exceeds the normal absorptive capacity of the small intestine (10–20 mg/day [2]), leaving unabsorbed iron which is available to colonic bacteria [36,38]. Traditional oral iron supplements are poorly tolerated and may exacerbate intestinal inflammation [39,40]. *Ferric maltol* (Feraccru^®^) contained 30mg of elemental iron per tablet. It is a new oral iron treatment containing a stable complex of ferric iron (Fe^3+^) with maltol which allows ferric iron to be absorbed by enterocytes without exacerbating disease severity in IBD patients with iron deficiency [41]. Our study suggests that FM exerts different effects on the microbiome too.

We undertook the first investigation of the effect of ferrous sulphate and ferric maltol on the human faecal microbiome. This was a pragmatic study that sampled patients with iron deficiency anemia who were being treated with ferrous sulphate and patients with quiescent inflammatory bowel disease receiving ferric maltol. At baseline, the taxonomy summary for stool at phylum level (Figure 6C) was similar in the two patient groups. Beta diversity analysis (Figure 6B1,B2) showed no effect arising from the iron supplement. The main factors that influenced the beta diversity were the patient ID and the diagnosis (IBD vs. non-IBD): thus, the presence of IBD had a greater impact on the beta diversity than iron supplementation.

Deeper taxa analysis showed no significant difference when FM samples (before and after treatment) were compared together, suggesting that none of the taxa were influenced by the drug. However, when comparing patients before and after FS treatment, the abundance of many genera changed significantly (Figure 6C,D). These data suggest that the unabsorbed ferrous preparation may contribute to a dysbiosis that does not occur with the sequestered ferric form of supplementation.

Murine models of IBD offer an opportunity to investigate bacteria and their pathways implicated in IBD and host–microbiota responses to treatments [42]. However, dysbiotic microbiota can induce murine colitis [43,44]. Nevertheless, currently, there is limited knowledge concerning the degree of similarity between the human and mouse gut microbiota particularly at the deeper levels of taxonomy such as genera and species [44]. Changes in microbiota are thought to be a major factor in human illnesses such as inflammatory bowel disease [45,46]. Some studies have shown significant results indicating that a subset of CD and UC samples contain abnormal gut microbiotas, characterised by depletion of commensal bacteria, particularly members of the phyla Firmicutes and Bacteroidetes and an increase in Proteobacteria [47,48].

The limitations of our human study arise from its pragmatic design. The patients who received ferrous sulphate had iron deficiency anemia from a range of causes. Patients receiving ferric maltol all had clinically quiescent inflammatory bowel disease. This reflects the license of ferric maltol. We did not have the resources to conduct a trial of the two preparations in a single cohort of patients. If, in the future, clinical trials comparing the efficacy of these different iron preparations are performed, then the impact on the microbiome could be further assessed. However, this weakness is partially off-set by the murine studies, the results of which support those in the human work.

## 5. Conclusions

The data in this study have shown differential and unique influences of different iron diets upon murine models of colitis and colitis-associated microbiota as well as in IBD patients. The data supports the use of ferric maltol to manage iron deficiency in IBD and minimise the risk of side effects and dysbiosis in these patients.

## Data Availability

Data available in Supplementary Files.

## Supplementary Materials

The following are available online at https://www.mdpi.com/article/10.3390/nu13072269/s1.
