# Supplementary material for: Oral Ferric Maltol Does Not Adversely Affect the Intestinal Microbiome of Patients or Mice, but Ferrous Sulphate Does
Source: Nutrients. 2021 Jun 30;13(7):2269. doi: 10.3390/nu13072269 (PMC8308237; doi:10.3390/nu13072269)
Supplement: Supplementary file 1 [file nutrients-13-02269-s001.zip › nutrients-1281557-supplementary.pdf]

Supplementary Tables

Table S1: IBD patients who received ferric maltol supplementation. Hb (haemoglobin), CD (Crohn's disease), UC (Ulcerative colitis), SCCAI (Simple Clinical Colitis Activity Index), HBI (Harvey Bradshaw Index), Transferrin Saturation reference range (20%-50%) (41), Ferritin (12-300 ng/ml male, 12-150 ng/ml female).

Sample ID	Sex	Age	Ethnicity	Type anemia	Hb	Ferritin	Transferrin saturation	Iron supplement	HBI	Montreal	SCCAI	Diagnosis	Calprotectin
FM1	M	54	White	IDA	129	16	11%	ferric maltol	3	A2L4B2	N/A	CD	29
FM3	M	46	White	IDA	123	7	5%	ferric maltol	2	A3L2B1p	N/A	CD	61
FM5	M	47	White	IDA	105	12	NA	ferric maltol	0	A2L3B2	N/A	CD	118
FM2	F	63	White	IDA	89	11	12%	ferric maltol	2	A2L2B1	N/A	CD	263
FM4	F	39	White	IDA	96	6	NA	ferric maltol	N/A	E3	1	UC	407
FM6	F	30	White	IDA	93	10	5%	ferric maltol	0	A2L3B1	N/A	CD	68

Table S2: IDA patients who received ferrous sulphate supplementation. Hb (hemoglobin), Hb 1m (after one month), OGD (Esophagogastroduodenoscopy), GU (gastric ulcer), GORD (Gastro-oesophageal reflux disease), NSAID (nonsteroidal anti-inflammatory drugs).

Sample ID	Sex	Age	Ethnicity	IDA	Hb	Hb 1m	Iron supplement	OGD	Colonoscopy	Diagnosis
367	F	48	White	IDA	10.8	N/A	ferrous sulphate	GU	N/A	GU
384	F	71	White	IDA	10.6	11.2	ferrous sulphate	Normal	2x polyp low grade	2X (1cm) polyp adenoma
385	F	79	White	IDA	11.6	12.1	ferrous sulphate	GORD	N/A	GORD
389	F	84	White	IDA	9.5	11	ferrous sulphate	Normal	Cecal Cancer	Cecal Cancer
412	F	77	White	IDA	10.9	12.5	ferrous sulphate	Normal	Normal	None
415	F	57	White	IDA	10.3	11.1	ferrous sulphate	GU	Normal	GU, NSAID
355	M	72	White	IDA	11.8	12.6	ferrous sulphate	Normal	N/A	None
388	M	68	White	IDA	11.2	12.4	ferrous sulphate	GORD	N/A	GORD
392	M	67	White	IDA	10.7	N/A	ferrous sulphate	GU	N/A	GU
401	M	70	White	IDA	11.5	N/A	ferrous sulphate	Normal	N/A	None

Table S3: Detail of taxa differential analysis results, including p values and adjusted p values

	Control-d1_vs_FSS-d10_Phylum				
	baseMean	log2FoldChange	pvalue	padj	Upregulated
Verrucomicrobia	1883.117817	-7.67714635	3.15E-19	2.20E-18	Control-day1

	Control-d1_vs_FMS-d10_Order				
	baseMean	log2FoldChange	pvalue	padj	Upregulated
Lactobacillales	3645.51595	2.001704613	0.00262042	0.039306262	FMS-day10

	Control-d1_vs_control-d10_Genus				
	baseMean	log2FoldChange	pvalue	padj	Upregulated
g__Bacteroides	3054.422974	-11.94146817	3.60E-66	5.40E-65	Control-day1
g__Lactobacillus	1102.399724	-3.336648706	8.33E-07	6.25E-06	Control-day1
g__Turicibacter	729.9449547	3.033064672	0.00057368	0.002386485	SC-day10

	FSS-d10_vs_FMS-d10_Genus				
	baseMean	log2FoldChange	pvalue	padj	Upregulated
Akkermansia	2365.128426	-8.006601732	7.20E-19	2.30E-17	FMS-day10
Bacteroides	3686.565685	-3.190089269	1.75E-05	0.000280467	FMS-day10
Ruminococcus	630.0125876	-2.144243411	0.00013511	0.001441211	FMS-day10
Lactobacillus	4427.936757	-2.364872921	0.00030868	0.002469413	FMS-day10

Butyricicoccus	150.0171607	-3.276386892	0.00099355	0.006358712	FMS-day10
Anaeroplasma	2684.707665	3.187947881	0.0020618	0.010996269	FSS-day10
Turicibacter	105.5009155	3.275490646	0.00254451	0.011632042	FSS-day10

Control-d1_vs_control-d10_Genus

	baseMean	log2FoldChange	pvalue	padj	Upregulated
Butyrivibrio	186.4276302	7.472710466	9.96E-10	4.78E-08	post-FS
Megamonas	499.1881487	6.945017554	1.32E-07	3.17E-06	post-FS
Megasphaera	56.95358321	5.745317503	4.11E-06	6.58E-05	post-FS
Lactobacillus	188.5541078	4.651070431	2.95E-05	0.000354097	post-FS
Acidaminococcus	22.02842168	4.813593187	0.00010608	0.001018403	post-FS
Prevotella	29.65707943	4.647893838	0.00016314	0.001305124	post-FS
Unclassified	18.19416858	-4.067673771	0.00032026	0.002196089	pre-FS
Turicibacter	42.64692706	-3.161764142	0.00218814	0.013128841	pre-FS
Dorea	348.8943072	-2.102518611	0.00258127	0.013766765	pre-FS
